# Global Breast Cancer: The Lessons to Bring Home

**DOI:** 10.1155/2012/249501

**Published:** 2011-12-05

**Authors:** Silvia C. Formenti, Alan A. Arslan, Susan M. Love

**Affiliations:** ^1^Department of Radiation Oncology, New York University School of Medicine, New York, NY 10016, USA; ^2^New York University Cancer Institute, New York, NY 10016, USA; ^3^Departments of Obstetrics and Gynecology and Environmental Medicine, New York University School of Medicine, New York, NY 10016, USA; ^4^Dr. Susan Love Research Foundation, 2811 Wilshire Boulevard, Suite 500, Santa Monica, CA 90272, USA; ^5^David Geffen School of Medicine at UCLA, Los Angeles, CA 90095, USA

## Abstract

Breast cancer is the most common cancer affecting women globally. This paper discusses the current progress in breast cancer in Western countries and focuses on important differences of this disease in low- and middle-income countries (LMCs). It introduces several arguments for applying caution before globalizing some of the US-adopted practices in the screening and management of the disease. Finally, it suggests that studies of breast cancer in LMCs might offer important insights for a more effective management of the problem both in developing as well as developed countries.

## 1. Introduction

Breast cancer is the most common cancer affecting women. Globally, about 1.4 million new cases are diagnosed each year [1]. Breast cancer incidence varies considerably throughout the world; age-standardized incidence is about 4-fold higher in high-income countries in North America and Western Europe compared to countries with the lowest per capita income [2]. A strong correlation between age-standardized incidence of breast cancer and the average gross domestic product per capita can be demonstrated [3]. However, in many low- and middle-income countries (LMCs), incidence is increasing at a faster pace than in developed countries, where the incidence is already high [4].

Several hypotheses for this rapid raise have been proposed. The most common explanation regards the rapid spreading of “westernization” of diet and lifestyle [4], characterized by switching to foods that have a high-energy density while decreasing physical activity, resulting in higher rates of obesity. Diet changes may also result in loss of protective factors associated with traditional diets. For example, westernization of diet in Japan and Japanese immigrants to the US is associated with marked increase of breast cancer incidence and mortality [5]. Changes in women's reproductive patterns, often as a result of improved socioeconomic conditions, include the occurrence of earlier menarche and delayed or reduced parity, each recognized as additional risk factors of breast cancer [6]. Breast cancer in Singapore's women exemplifies the impact of these changes as their incidence of breast cancer is rapidly approaching that seen in Europe [7], a worrisome sign of the looming epidemic of breast cancer in other rapidly developing countries such as China and India. In addition, longer lifespan, better policies for reporting disease diagnosis and the introduction of screening, at least in some countries, all contributed to the increasing breast cancer incidence in LMCs.

## 2. Differences in Epidemiology and Clinical Presentation of Breast Cancer in Low-Income Countries: The Example of Sub-Saharan Africa

Breast cancer in LMCs differs both in incidence and clinical presentation from its counterpart in Western countries. Protective factors like delayed menarche, early- and multiparity, and protracted breast-feeding all contribute to reduce the risk of breast cancer in LMC, as demonstrated by its lower incidence in Africa [8].

Nevertheless, breast cancer is the second most common cancer among women in Sub-Saharan Africa, accounting for 16.8 percent of all female cancers: an estimated total of 48,600 cases occurred in Sub-Saharan Africa in 2002 [9].

Noticeably, while less frequent, it is more fatal than breast cancer in Western countries. The analogy with breast cancer in African American women, also less frequent in incidence and more fatal, has led to the hypothesis that inherited factors associated with African heritage could contribute to this outcome. Since most of the colonial slave trade involved Sub-Saharan populations, Fregene and Newman [10] reviewed the published information to explore correlations between the characteristics of breast cancer in Sub-Saharan Africa and in the United States, among women of African American descent [11]. Despite the existing barriers to obtain accurate information in countries without a well-organized tumor registry system, consistent descriptors of Sub-Saharan breast cancer emerged from this analysis: a typical incidence between 35–45, later clinical stage, and higher mortality. While a complete histopathological characterization of these tumors is often lacking because of limited health care resources, Ikpatt et al. examined several hundred breast cancer specimens from Nigeria and compared them to tumors from Finnish women: a pattern of higher proliferative rate, necrosis, and nuclear atypia was detected among the Nigerian specimens [12]. Similar conclusions were reached in a review by Ly et al., who noticed that reported tumors were mostly invasive ductal carcinomas with aggressive characteristics: grade III histoprognosis, absence of hormonal receptors, or HER2 expression, with nearly half of these tumors classifiable as triple negative [13].

Among 1255 breast cancer women treated between 1999 and 2006 at Gezira University in Central Sudan, 74% were premenopausal at the time of disease presentation, which was predominantly at stages III and IV (60.7%). Among the few patients whose tumor specimens had undergone assessment of hormonal receptors expression, only in 20% of the cases, they were found to be positive [14]. A similarly low rate of ER/PR positive tumors was found in a study of breast cancer patients diagnosed in Nigeria [15].

A recent report analyzing 57 breast cancer specimens from Ocean Road Cancer Institute (ORCI) in Dar es Salam found ER-PR-histology in 49%. The same study reported that 90.7% of the 356 patients staged presented with stages III and IV disease [16].

While sociocultural factors are likely to underlie delay in diagnosis, some evidence also suggests a more rapid course of the disease in these populations, associated with the prevalence of a more aggressive breast cancer phenotype, as confirmed by a more frequent incidence of the triple negative subtype, compared to that diagnosed in developed countries. Elucidation of etiology and predisposing factors to the disease in Sub-Saharan women is likely to contribute to the understanding of breast cancer in African-American patients and, possibly, in other subsets of women with triple negative disease.

Furthermore, the hypothesis of an infectious etiology of breast cancer in Africa cannot be excluded. It was introduced by the evidence of a relatively increased incidence of breast cancer in men, compared to that observed in non-African countries where it is a rare disease in males (*P* < 0.05). For instance, the male/female ratio in Tanzania is 1 : 14 (0.071), similar to the overall ratio does in the majority of Sub-Saharan African countries (0.0143; CI = 0.0317–0.877) [17].

In summary, although breast cancer incidence is lower in Sub-Saharan African countries than in developed countries, women tend to be diagnosed at a more advanced stage than women in the developed world and are more likely to die from it. Unfortunately, early detection of the disease is hampered by limited awareness and understanding of the disease even among health care professionals [18, 19].

In this regard, the experience of Sudan is instructive. Sudan first initiated a national cancer control program (NCCP) in 1982 in association with the World Health Organisation (WHO). This program focuses on prevention, early detection, and screening, improved diagnosis and treatment, and palliative care. Sudan has educational strategies for both the general public and medical professionals to dispel many of the misconceptions about cancer and promote early detection and referral of cancer patients. Radio was selected as an important medium in a country of low literacy rates. To raise awareness amongst women about the importance of self-examination for breast irregularities, a simple booklet was produced and widely distributed [20].

## 3. Progress in Breast Cancer in Western Countries: A Reality Check

Breast cancer is the most costly disease in the US because of its common incidence. About $16.5 billion are spent each year in the US on breast cancer diagnosis and treatment. When lost work productivity due to breast cancer for an additional $12.1 billion is added, and about $2.5 billion for breast cancer-specific peer-reviewed research is included, the expenditure exceeds $31 billion/year [21].

A reasonable assessment of the results from this investment requires multiple measures of outcome with parameters that span from a reduction in disease-specific mortality to improvements in quality of life after diagnosis. Breast cancer mortality is decreasing, and Berry et al. [22] have estimated that during the past ten years it has approximately decreased 1% per year due to a combination of screening and adjuvant treatment, both equally contributing to this progress.

This pace of the yearly reduction of mortality, however, has been questioned. It remains unclear whether this small incremental progress could be sustained until eradication of the disease, and it is certainly much too slow to be acceptable to survivors, advocates, and many health care professionals caring for breast cancer patients. In addition, the mortality reduction has not been universal, with less progress in women under 50 and in African-American and Hispanic women. Moreover, the common use of mortality-over-incidence ratio, as a measure of success in eradicating a disease, may overestimate the objective progress in breast cancer. This is due to the fact that much of the increased incidence pertains to “trivial” cancers, mostly detected by mammography screening, unlikely to represent a fatal hazard [23, 24].

Evidence has also emerged that at least some of these mammography-detected cancers may spontaneously regress. A change in the routine screening protocol in Norway permitted this observation. In 1996, the Ministry of Health and Care Services initiated the Norwegian Breast Cancer Screening Program, in which women between the ages of 50 and 64 were invited to undergo a two-view biennial screening mammography program. The women in this study who make up the “screened” group thus underwent a first round of screening mammography in 1996-1997, a second round in 1998-1999, and a third round in 2000-2001. Women (age range 50–69 years) who would have been invited to participate in the screening program between 1992 and 1997 had the Norwegian Breast Cancer Screening Program existed, then, were studied as a “control group.” These women only underwent one screening mammogram at the end of their six-year period compared to the three mammograms the women in the screened group underwent. The investigators excluded carcinoma *in situ* (DCIS) and concentrated only on invasive carcinoma. The screened group had 22% more diagnoses of invasive carcinoma than the group that only received one screening test at the end of a six-year period, suggesting that approximately 1/5 of breast cancers may regress spontaneously [23].

In summary, the breast cancer problem remains mainly unsolved in Western countries, much in need of more effective solutions if eradication is the target. Moreover, differences of the disease in LMCs further temper any enthusiasm to export our existing screening and treatment protocols.

## 4. Differences in Menopausal Status at Presentation

The incidence of breast cancer in Caucasian women in the Western World peaks after menopause, whereas in the rest of the world, it peaks before menopause [25]. A recent analysis suggested that at least two types of breast cancer exist, based on their clinical course: rapidly progressing (diagnosed before menopause, estrogen receptor (ER) negative, with a poor prognosis and high mortality rate) and slowly progressing (diagnosed after menopause, ER positive, with a good prognosis and low mortality rate) [26, 27].

For example, nulliparity and obesity decrease breast cancer risk in younger women but is associated with increased risk in older women. High-grade, aggressive tumors are more common in younger women, whereas low-grade tumors are more common among the elderly, with bimodal peak frequencies at ages 50 and 70, respectively. Curves depicting the annual hazard of breast cancer death for women with early-onset and late-onset tumors are shaped differently and move in opposite directions [28]. In addition, mammography screening and neoadjuvant chemotherapy seem to have different effects in women with early and late-onset tumors. Taken together, these qualitative age interactions may indicate that early- and late-onset breast cancers are likely to be different diseases, derived from different pathways [28].

Applying these observations to the pattern of breast cancer observed in LMCs suggests that the rapidly progressing type might be predominant in LMCs, as it is in young Hispanic women [29], as opposed to the slowly progressing, late-onset type more commonly observed in Caucasian women from developed countries.

This hypothesis has several implications.

1. Breast cancer in LMCs, young, and non-Caucasian women might have a different etiology than that occurring in women from the Western world.
2. Higher breast cancer mortality rates in LMCs young and non-Caucasian women are only partly due to patterns of care and lack of screening.
3. Current approaches to screening may not be as effective in these groups and should be reexamined from this vantage point.

## 5. Mammography Screening: Is the Western World Experience Feasible in LMCs?

In many Western countries, mammography screening for early detection of breast cancer has become a standard of care and the most common form for disease detection. Despite its widespread use, mammography is far from being a perfect means of early detection. Several limitations have been recognized, particularly if mammography screening is proposed as a strategy for addressing the global breast cancer problem.

### 5.1. False Positive Results

In the US, an average of 11 percent of screening mammograms is read as abnormal and necessitates further diagnostic evaluation [30]. Breast cancer is found in about 3 percent of women with an abnormal mammogram (representing 0.3 percent of all mammograms). Fletcher and Elmore demonstrated that, on average, a woman has about a 10.7 percent chance of a false positive result with each mammogram [31]. A woman's risk of having a false positive mammogram increases over time with repeated screenings. Elmore et al. [32] estimated that, after 10 mammograms, about half of women (49 percent) will have had a false positive result, which would have led to an unnecessary needle biopsy or an open biopsy in 19 percent of screened women. Conversely, the false negative rate of 20–26% [33] contributes to a false sense of security in some women and it may result in delaying their time to diagnosis.

A Cochrane systematic review of six clinical trials involving half a million women concluded that mammography screening resulted in an absolute risk reduction for mortality of 0.05% and was associated with an absolute risk of overdiagnosis of 0.5% [34]. This means that for every 2,000 women invited for screening over a 10-year period, one woman will have her life prolonged, whereas 10 healthy women will be overdiagnosed and treated unnecessarily [34]. These numbers are sobering and should be carefully considered before proposing to introduce mammography in LMCs.

### 5.2. Age Distribution and Breast Cancer Screening

Mammography is most effective for detection of slow-growing tumors in postmenopausal women, after age 50. In Western countries, mammography screening in women aged 40–49 years has failed to result in a significant reduction of breast cancer mortality [35, 36]. A similar lack of effect could be foreseen in LMCs, where the majority of women are under the age of 50 and breast cancer tends to occur earlier. Interestingly, the early mammography screening trials before the introduction of adjuvant systemic therapy actually demonstrated an increase in breast cancer mortality rates among women who were aged less than 50 years at the start of those trials [37]. This has been referred to as a “mortality paradox” potentially associated with the inadequate treatment of the early-onset cancers or, possibly, a surgery-induced angiogenesis in breast cancer [38]. Since adjuvant treatment options are often lacking or are not affordable for many women in LMCs, it is likely that the “mortality paradox” would be observed in LMCs as well.

### 5.3. Ethnic and Biological Differences

Ethnic and biological differences may further reduce the benefit of mammography. For instance, women in many Asian LMCs typically have smaller breast volume and relatively dense parenchyma compared to Caucasian women [39], both characteristics unsuitable for screening mammography.

### 5.4. Social and Cultural Barriers

Misconceptions associated with biopsies triggered by screening mammography could be particularly harmful in some LMCs, where a low level of knowledge about cancer and the meaning of screening prevail. For example, after a breast biopsy women and their families could erroneously be “labeled” as cancer carriers (even when the results are negative), resulting in social stigmatization and ostracism with dramatic psychosocial and economic consequences. Serious ethical concerns emerge with mammography screening in LMCs if followup and treatment are not made available or affordable for the majority of women.

### 5.5. Harm-to-Benefit Ratio

As mammography screening programs are promoted around the world, the common tendency is to emphasize the benefits and deemphasize the harms [40]. The harms of mammography screening consist of false-positive findings resulting in anxiety for the woman and her family, lack of affordable treatment and followup, overdiagnosis (detection of early lesions like DCIS, most of which would never have clinically surfaced in the individual's lifetime), and false-negative exams. The harm-to-benefit ratio should be carefully considered prior to implementation of mammography screening in LMCs. In the US, most screened women tend to overestimate the benefit of a screening procedure [40], an issue amply debated in the UK and other European countries [41].

### 5.6. Cost-Effectiveness

Mammography screening programs are expensive to initiate and sustain. The WHO Commission on Macroeconomics and Health criteria has proposed that a health intervention can be considered cost-effective if it achieves savings of one disability-adjusted life year for less than three times of a country's GDP [3, 42].

To date, very few studies addressed the cost-effectiveness of mammography screening in developing countries. Two recent analyses of breast screening concluded that it is not cost-effective in countries like China [43] and India [44].

## 6. Western Treatment Model: Treating the Many to Benefit the Few

Therapeutic options derived from existing experiences may also be as disappointing. For instance, in Western experience, the tumors most frequently detected by mammography are those <2 cm in size (tumor size T1), often treated by breast conservation. In this subset, if no cancer cells are found in the lymph nodes, survival 8 years after breast conservation surgery and radiation is about 86%. Attempts to further improve this rate require treating all to benefit only a few. Five years of tamoxifen treatment increases the overall survival at 8 years from 86% to 93%; adding chemotherapy results in a further gain of 2%, to a total of 95% at 8 years [45]. Otherwise stated, 86 of 100 women with early breast cancer will be alive and free of detectable disease 8 years after surgical excision, regardless of any additional hormone therapy or chemotherapy. If tamoxifen is added, 93 of 100 women will be alive at 8 years. Because 86 of those would have been free of disease whether they were treated with tamoxifen or not and 7 would have died, nevertheless, the real benefit is confined to 7 women. As a result, 100 women are treated to really benefit only 7. The data is even more sobering when chemotherapy is added, for every 100 women receiving chemotherapy, only 2 will derive benefit from this additional treatment. Since chemotherapy can cause significant side effects, deciding its use for this subset of patients is difficult in Western countries and even more so in LMCs.

## 7. Breast Cancer in LMCs: A Different Approach

Instead of translating Western experience to LMCs, we propose that breast cancer in LMCs represents a unique opportunity to understand this disease and, at the same time, the occasion to objectively reassess our progress. For instance, original solutions to effectively interfere with recurrence of breast cancer in LMCs have already emerged [46, 47].

It is likely that more of these cost-effective approaches will emerge from local stakeholders, aware of the complex reality of health delivery and care in their country. For example, handheld ultrasound devices for on-site diagnosis of palpable lesions could be operated by ancillary health personnel. Well-designed prospective studies could identify variables associated with successful detection and result in an algorithm that enhances accuracy in distinguishing malignant masses from benign ones. Insights about etiology could be derived by studying associations of specific changes in lifestyle and diet with the incidence of the disease. In addition, new lessons about hospice and palliative care can be learned [48]. The results of these studies could result in development of specific guidelines for LMCs [49] and open the way for novel interventions to be tested worldwide.

Since locally advanced breast cancer remains the most common presentation of the disease worldwide, detection and downstaging programs [50] focused on effective treatment may demonstrate more cost-effective in LMCs than a campaign for screening and early detection. Importantly, in LMCs that have not introduced mammographic screening for early detection, it is more likely for incidental cancers or trivial cancers to remain undiagnosed and not contribute to enhance the denominator of incident cancer cases. In such a setting, an intervention that successfully reduces breast cancer mortality is easier to assess, since the reduction of breast cancer mortality is a more reliable parameter to follow progress. Introduction of novel therapeutics could reflect a targeted approach, by limiting treatment to women who are highly likely to derive survival advantage from their response, as already done in other diseases [51, 52].

Recent high-level meetings on noncommunicable diseases, as part of the 66th session of the United Nations General Assembly, identified several priorities including raising the awareness about cancer in LMCs and to make the best use of available resources to strike a balance among prevention, detection, and therapy [53].

## 8. Summary

In conclusion, the emergence of evidence that breast cancer in LMCs is distinct from its counterpart in Western countries discourages the introduction of widespread programs of population screening. In settings with limited resources, focusing on expensive programs to identify women who are at risk will inevitably deprive resources to treat and potentially cure the affected individuals. Similarly, therapeutic intervention should reflect the social and cultural specificity of the country in question, a task best left to indigenous health care providers. Importantly, LMCs could be spared by the repetition of experiences that mainly failed in Western countries.

Addressing the global challenge of breast cancer requires a dispassionate reassessment of the progress Western countries have made, with a critical analysis of the current practices. In fact, the opportunities of studying breast cancer in the rest of the world may provide us with much needed insight about what we have missed, despite so much human and financial investment.
